# Embedded dyads: How the personal network relates to linguistic accommodation

**DOI:** 10.1371/journal.pone.0357457

**Published:** 2026-09-21

**Authors:** Guillaume P. Fernandez, Eric D. Widmer, Robin Dodsworth

**Affiliations:** 1 Department of Sociology, University of Geneva, Geneva, Switzerland; 2 LIVES Centre, Swiss Centre of Expertise for Life Course Research, University of Geneva, Geneva, Switzerland; 3 Department of English, North Carolina State University, Raleigh, United States of America; Instituto Tecnologico Autonomo de Mexico, MEXICO

## Abstract

Language serves as a key medium through which individuals express and negotiate social belonging. By observing individuals’ behavior within an interaction, we can access the relational stance they adopt and infer the social relationships that link the two interactants. This process is particularly studied within the framework of the Communication Accommodation Theory (CAT). According to CAT, speakers tend to align linguistically with their interlocutors to signal social proximity. Yet, most analyses remain focused on the psychological and dyadic levels, without considering the broader social influences that may shape linguistic choices. In this paper, we argue that considering the relational structure surrounding both interactants is crucial. In particular, we hypothesize that the position of the interlocutor within the speaker’s personal network is a key factor driving linguistic behavior. To address this, we conducted an exploratory empirical investigation with 21 young adults who recorded themselves in an average of three interactions each (resulting in a total of 71 discussions). Their personal networks and linguistic data were both collected. Linguistic similarity was operationalized via the Manhattan distance of intensifier frequency between the respondent (ego) and the interlocutor (alter). We used Generalized Estimating Equation models to test our hypotheses. Results indicate that speakers tend to align linguistically with their interlocutors when the latter occupy a central position within a compositionally homogeneous cluster—that is, when the alter is embedded in a structurally dense subgroup composed of individuals originating from the same social domain.

## Introduction

Linguistic behavior is a key marker of identity, enabling individuals to position themselves within social spaces and express social belonging. One primary way people use language is through convergence. Aligning one’s linguistic behavior with that of an interlocutor signals social proximity and affiliation. In the early 70s, the Communication Accommodation Theory (CAT; [1]) became an influential theoretical framework for research on speakers’ shifting linguistic choices within and between interactional contexts.

From its early development through its later extensions [2], CAT has centrally involved a *relational* view of language, focusing on the social relationship between the speaker and interlocutor [3]. Managing social relations is, in fact, one of the main motives for accommodation [4]. Indeed, linguistic adjustment allows individuals to highlight and negotiate group membership [5]. By way of convergence, individuals can show social belonging to the reference group [6], whereas divergence can reflect and reinforce social distinctions between interlocutors. Accommodation is widely regarded as a key mechanism for reducing social or relational distance in interpersonal communication. Emphasizing similarities between individuals helps diminish uncertainty about others and fosters a sense of connection [3].

As Côté and Clément (1994) argued, the choice to converge in a situation is deeply rooted in the substance of the relationships that link the two interactants [7]. Although CAT primarily addresses dyadic interactions (e.g.[8],), it often neglects the reality that each conversation partner (alter) holds a distinct structural role within the speaker’s (ego’s) wider social network. The degree to which an alter is embedded or peripheral in this network may shape the dynamics of accommodation. We propose that accommodation is not only dyad-based, but also shaped by the alter’s position in the ego’s personal network. In particular, structural features such as embeddedness, clustering, and composition may influence how convergence unfolds.

While CAT is a relational framework, research on linguistic accommodation traditionally has not employed statistical methods specifically designed to analyze relational processes. Social network analysis (SNA) holds potential to investigate new hypotheses within the CAT framework [9]. Using SNA to study language is not new. It has been used in sociolinguistic research since the seminal work of Milroy (1987), and more recent studies continue to explore the interplay between social networks and language (e.g.[10–15],). This study is, to our knowledge, the first to use social network methods to address how accommodation is relational.

This contribution is organized as follows. We first present the tenets of CAT and how it can relate to the relational processes. This will allow us to propose hypotheses about how the accommodation process is linked to the relationships that hold between the respondent and the different members of their personal networks while taking into account the embeddedness of the dyad. To tackle this, we conducted an exploratory empirical study with a purposive sample of 21 French-speaking college students from vocational schools in Switzerland, aged 20–30. The participants were enrolled in diverse fields, including management, architecture, design, health and nursing, nutrition, midwifery, social work, music, engineering, information technology, and landscaping. Each participant recorded an average of three interactions. Seventy-one different interactions between the respondents and the members of their personal networks were recorded and analyzed. Although the analyses should be considered exploratory, given the number of participants, the sample is unique in that it is the first to bring together data from personal networks and multiple face-to-face interactions from the same respondent. It offers an unmet opportunity to study how accommodation unfolds in dyadic interactions and how these interactions may be constrained by larger social structures. The statistical analyses were performed with General Estimating Equation (GEE) models to account for the nested structure of the data. Results and limitations are discussed in the final sections.

### Communication accommodation theory

A foundational premise in the field of sociolinguistics is that language is indexical [16]. That is, linguistic choices hold social meaning and allow speakers to position themselves in social space [17]: a group differentiates itself through the particular use of a linguistic form; this form is perceived and evaluated by others; it becomes associated as typical of that group and ultimately acquires the social meaning that was initially attributed to the group as a social entity. Then, every social practice is perceived as representative of a group [18], and the individual therefore proceeds to an evaluative phase to decide whether or not to use this indexical practice.

It is within this process that Communication Accommodation Theory (CAT) [1,19] is seminal in explaining linguistic behavior in interaction, because dyadic encounters are conceptualized as a micro-expression of intergroup relations [20,21].CAT predicts that individuals either converge, aligning their language with their interlocutors to signal connection, or diverge, emphasizing differences to assert distinct identity. Convergence is often motivated by a desire for social approval, while divergence highlights dissimilarity, which can negatively impact relationships, a phenomenon Gasiorek (2016) calls the “dark side” of adaptation [22]. CAT suggests that the more an individual identifies with or feels connected to a valued ingroup, whether based on ethnicity, family, sexual orientation, or other attributes, the more they will seek to emphasize that social identity through their communication [23]. Beyond the motivational aspect for convergence, accommodation can also have a mediating or indirect effect on attitudinal outcomes [24], such as emotions, relational solidarity, and trust.

### Extending CAT: from dyads to ‘embedded dyads’

CAT is mainly conceived at the dyadic level. However, dyadic behaviors and attitudes are embedded within broader network substructures [25]. In particular, dyads are parts of complex chains of emotional, cognitive, and instrumental interdependencies [26,27]. These structuring patterns posit that dyads cannot be analyzed as stand-alone units with their own self-organizing forces. This statement implies that convergence processes are influenced not only by the direct relationship between ego and alter but also by their integration in the overall network. In other words, social identification is driven by both individual interactions and the broader relational dynamics within the group.

Before moving forward, let us note that the ‘actual’ or objective structuration of social relationships is not the sole driver of behavior, but instead, it is acknowledged that the subjective perceptions of networks are often more informative than objective structures [28]. Cognitive networks provide insight into how individuals exercise social agency in daily life [29,30]. Individuals tend to imagine relationships that, in reality, do not exist, either by creating nonexistent connections or by not perceiving existing ones [31]. The perception of reality, in turn, motivates actions [32] and has real consequences for cognitive and emotional states [33,34].

A clear cognitive map of social relationships simplifies understanding others’ behaviors and expectations, allowing individuals to act appropriately while minimizing the risk of social costs [35]. Network configurations that enable individuals to develop a clear mental and social representation of their interlocutors in the environment should help them navigate different social contexts and adjust their linguistic behavior accordingly [35]. By mental and social representation, we mean the ability to situate the people that constitute the personal network into well-identified and distinct social groups.

The overlapping and repeated interactions with a limited group of individuals make behaviors and attitudes more predictable and facilitate the formation of clearer and stereotypical mental representations of each member [13,36–38]. This is also supported in the work of Brashears and Quintane (2015), who provide empirical evidence that people tend to recall their networks in terms of groups and triads, rather than dyads [39]. That is, people more easily map their social relations in aggregated and clustered ways rather than as the mere sum of dyadic interactions. This perceived structure consequently offers opportunities to guide decisions and behaviors [39].

More precisely, early sociolinguistic literature (e.g., [10]) emphasized the role of clusters, zones with strong internal connections and limited external ties, in assessing a network's capacity to enforce norms [10,13,38]. The small and dense subsets of a network increase the likelihood of overlapping interactions among similar people and therefore create a clear mental representation.

### Hypotheses

Together, this suggests that relational factors outside the direct dyad are expected to influence accommodation. While CAT has extensively modeled dyadic convergence, it has yet to account for how this social unit may be conceptualized as belonging to larger relational structures and, therefore, how an interlocutor’s position within it might be related to this process. We argue that CAT should be extended to incorporate structural features surrounding the interlocutors. Their position in the speaker’s network, and how they belong to clusters, in terms of structure and composition, and more extensively, the intersection of both, matters.

### The relational structure surrounding the alter

Among structural factors expected to influence accommodation, one that almost systematically stands out is the position of the alter in the network and their relational embeddedness. Two reasons can be invoked to explain why the embeddedness of the alter within clusters of the personal network is expected to be related to accommodative behavior: Alters highly embedded within a subgroup are likely to be seen as representative of such subgroups and therefore also help the ego to develop a clearer mental representation of their social identity and group belonging [37]. In this sense, alters who are well embedded in clusters should be easily identifiable and therefore help guide the behavior of the speaker.

In addition, speakers are more likely to accommodate to desirable alters, and their desirability depends in part on their prestige [40]. In the social network analysis tradition, prestigious individuals are the ones occupying a central position within the network [25,41].

Recent empirical evidence suggests that individuals are likely to identify more strongly with central, well-connected, and embedded alters, and that this fosters a greater perception of similarity [15]. It has also been investigated as a driver of language use (e.g., 10, 40, 41). In the context of accommodation, we expect individuals to linguistically align with people who are central and structurally well-embedded within the speaker’s network [25]. Explicitly, we hypothesize that:

H1: The more an alter belongs to a structurally dense cluster of the speaker’s personal network, the more the speaker is likely to accommodate that alter.

### Who is connected to the alter?

An essential insight from sociolinguistic research highlights the significance of not only the structural aspects of social networks but also their qualitative composition [42–45]. It should be related to the process of accommodation, as a homogeneous cluster limits exposure to diverse cultural backgrounds, and therefore helps the development of a clear mental representation of the alters.

Eckert (2000) observed that individuals adapt their language to align with the speech patterns dominant within their social networks [46]. This is also reflected in earlier work (e.g.[47], which shows that homogeneous networks lead to more homogeneous speech. Conversely, ethnic diversity within a network is linked to a broader range of linguistic repertoires and greater linguistic variability across contexts [13].

In this paper, composition is not constrained to personal attributes, such as gender, ethnicity, or class, but relies on a more sociological perspective: social domains, or social roles. Social domains (e.g., friends, family, colleagues) are related to linguistic behavior [15,48]. Individuals adapt their language to align with the specific social domain they are engaging with [48]. As a result, individuals are expected to adapt to their different social domains, as Mische and White [49, p. 702] argue: “distinct registers of speech appear for different social domains, such as family, business, church, or politics.” Dense friendship networks have been shown to predict some kinds of lexical similarity in written language [50]. Additionally, research shows that young adults tend to distance themselves from family members as they strive for autonomy and develop a unique sense of self [51]. By reinforcing their affiliation with peers [8], young adults are more likely to detach themselves from their parents and perceive them as less linguistically similar [15]. This transition to adulthood is linguistically marked by an “adolescent peak” [40], which translates into a deliberate shift in language use to signify this distancing from familial influences [8].

Therefore, if an alter belongs to a compositionally homogeneous part of the personal network, in terms of social domains, we hypothesize that:

H2: The more the alter is in a homogeneous cluster in terms of composition, the more the speaker is likely to accommodate to that alter.

### Structure and composition work together

Although we have argued that a dense network can be thought to be the kind of structure that will enhance the accommodation with each alter, the evidence in the literature brings some nuances, if the composition is not taken into account together with the structural density.

Clusters do not happen at random, but rather are formed around social roles [52]. That is, there is a social tendency for people of the same social role – or even social attribute –to connect and to create dense networks [25,51]. Although clusters are not expected to be formed around people of different backgrounds, it does frequently happen [52], especially when some ties cannot be formed merely due to the nonavailability of choices. People belong to larger social structures (e.g., geographical locations, workplace), which are not uniformly composed [53]. Therefore, some people may not be able to form ties with similar others only because they are not present in the direct social context.

Dense networks composed of varied roles would rather prevent the use of role-based behavior [54]. Indeed, in heterogeneous dense networks, where individuals interact across diverse social roles and backgrounds, there is a higher likelihood that people from different social contexts will meet. This increases the potential for social costs, as behaviors or language appropriate in one social role may be inappropriate in another. To navigate this complexity and avoid miscommunication or negative social judgments, individuals tend to adopt a neutral, role-independent language, rather than one tied to a specific group or social identity. This idea aligns with the fact that individuals interacting across varied groups or social backgrounds are more likely to use less localized forms of language [10]. The motivation here is primarily instrumental: using neutral language increases the efficiency of communication by reducing the risk of misunderstanding, especially when interlocutors do not share the same linguistic repertoire.

Beyond this instrumental rationale, a social identity dimension can also be considered. In diverse and overlapping social environments, individuals may avoid role-specific language not only to enhance communication but also to manage impressions and avoid being misidentified or seen as deviant. Thus, rather than tailoring their language to each specific role or group, people may prefer a consistent, neutral linguistic style that minimizes social risk and supports broader acceptance across contexts.

The first two hypotheses argue that, on one hand, a compositionally homogeneous network, and, on the other hand, a dense and localized network, matter. Nevertheless, we assume that, alone, these two aspects may only capture partial mechanisms and that the effect would be stronger if composition and structure were integrated. The interplay of both structure and composition of the alter's network neighborhood is then expected to be of importance in ego’s linguistic accommodation. Strong bonds that emerge through overlapping relationships and shared group memberships [36,55] increase the influence of alters on ego. In terms of language, preliminary research suggests that individuals tend to be less linguistically versatile across contexts when interacting with those who share the same social domain and are embedded in dense local supportive patterns [15]. When considering both structure and composition, we expect that:

H3: The more the alter belongs to a structurally dense and homogeneous cluster, the more the speaker is likely to accommodate that alter.

## Methods

### Ethical considerations

This research received ethical approval from the University of Geneva Ethics Committee (CUREG 2.0; approval number: CUREG-2022-06-63). Participants in the research project were informed, prior to participation, through an information sheet and consent form, about the nature and scope of the study. This included details on the individuals responsible for data processing and analysis, the type and extent of the data collected, and the purpose of the data processing and analysis. Participation was entirely voluntary, and participants were explicitly informed of their right to withdraw their consent at any time while completing the questionnaire, without incurring any disadvantage.

### Sample

The sample consists of vocational college students from the French-speaking region of Switzerland, specifically those enrolled in the Hautes Écoles Spécialisées de Suisse Occidentale (HES-SO). These students represent diverse fields, including health, design, engineering, architecture, management, music, art, and social work, across multiple cantons. Recruitment was carried out through professors, institutional directors, HR departments, and research centers within these institutions. The research team introduced the study in classrooms, explaining the data collection process. Interested students provided their email addresses to participate. We thus have a purposive sample, which might prevent population inference. However, the data is unique, as it is the first to collect, at this scale, both data on personal networks – with the most developed measurements of social relations – and discussions between the respondents and their members. Therefore, it provides an unprecedented opportunity to explore how accommodation may be related to network influence.

Data collection took place between October 2022 and the end of May 2024 and involved presenting the study to 80 classrooms. Of the 250 students who expressed interest, 161 completed the network survey, and 21 participants also provided recordings of multiple interactions. These 21 respondents, primarily aged 20–29 (92%) and predominantly female (80%), recorded an average of three discussions each, resulting in 71 dyadic encounters for analysis. Since only two respondents fell outside the 20–29 age range, we exclude respondent age from the analysis.

### The personal network

To investigate how respondents adapt to their interlocutors and explore the relational dynamics driving this process, we utilized social network analysis (SNA). The perceived personal networks of respondents were reconstructed in three steps [26].

The first step involved administering a Name Generator. Using the Family Network Method [56], respondents listed individuals who had a significant importance for them—positively or negatively—over the past six months. These individuals constituted the respondent's personal network (alters). The second step consisted of the Name Interpreter, in which sociodemographic information was collected for each alter. The third step was the Edge Interpreter, where respondents identified the emotional support provided by each alter and mapped perceived support exchanges within the network [29,57,58].

### Language: Intensifiers

The linguistic feature used to assess linguistic accommodation, in the present paper, is variation in the choice of French intensifiers, or words that adjust the intensity of the following adjective or adverb. For example, in the expression *very hot*, the intensifier *very* increases the degree of the adjective *hot*. Intensifiers can either amplify or tone down the following adjective or adverb. Intensifiers are a good choice for assessing linguistic convergence because the repertoire of intensifiers in a language is expected to undergo frequent and rapid change, with the result that several frequent intensifiers co-occur at any point in time. For example, in English, the intensifiers *really* and *very* have both been frequent for several decades, together with other intensifiers such as *so* and *pretty* [59–62]. Similarly, in a study of French intensifiers in Quebecois films, Vargas Alvares (2024) finds that while *bien* and *très* are overall the most frequent, *vraiment* and *tellement* are rising in frequency among younger speakers [63]. Intensifiers also carry strong but changing social associations, making them useful for identity or stance construction, expressing emotion, or marking social proximity [60,63–65]. In addition, intensifiers occur frequently in everyday speech, an important feature for the present analysis.

Social factors significantly influence the choice of intensifiers [66]. Age is a key determinant because the repertoire of intensifiers and their social associations changes rapidly. Some studies find that women use intensifiers more frequently than men [59,67], and in other cases, gender interacts with education [66]. As societies shift toward greater gender equality [67], the influence of gender must be considered alongside other intersecting variables such as age, socioeconomic status, geography, register, and discourse context [66,68]. Intensifiers fulfill both a grammatical function and a pragmatic function, with the latter being shaped by social factors and used to convey nuanced meanings. These attributes make them a valuable linguistic feature for exploring how social networks influence convergence.

### Measurements

Building on this linguistic and social framing, we now turn to the empirical measurement of convergence through intensifiers. To measure this, we retrieved the similarity of intensifier use between ego and each of the alters. To minimize the observer’s paradox [69], participants recorded themselves during natural conversations with up to five alters. These self-recordings are considered the “gold standard” for informal language studies [70].

We follow a similar procedure as the Language Style Matching operationalization (LSM [45],. The LSM is a measure that accounts for the frequency of varied parts of speech that belong to the function words category (e.g., pronouns, determiners, intensifiers, etc.). For each category, a percentage of the total word production is computed. Our approach slightly differs in the sense that it only focuses on intensifiers, and it does not capture the overall percentage of use of all the intensifiers merged as a single category. Rather, it calculates the relative frequency of each intensifier, which offers a more nuanced quantification of intensifier production. Every intensifier, including both amplifiers and downtoners, that modified an adjective or adverb was included. This represents a methodological difference between this analysis and previous sociolinguistic intensifier studies, which address only adjectival modifiers. The frequency of each intensifier was calculated per interaction, followed by pairwise comparisons between ego and each alter using Manhattan distance. Manhattan distance is a common measure for assessing linguistic matching in Natural Language Processing [71]. This measure quantifies linguistic similarity, with scores ranging from 0 (complete similarity) to higher values (greater dissimilarity) [72]. For example, if ego uses the same intensifiers at similar rates as the alter, the Manhattan distance is 0. Larger distances indicate significant divergence in intensifier usage. Finally, we reversed the scale to capture convergence rather than divergence.

To address H1, which stipulates the extent to which the accommodative behavior of the focal individual is related to how the interlocutor belongs to a structurally dense cluster, we use the clustering coefficient. The clustering coefficient captures the extent to which an individual’s neighbors are connected to one another, and is computed as the proportion of closed triads among all possible triads in the local network [38]. The clustering coefficient ranges from 0 (not integrated) to 1 (perfectly integrated). For example, if alter A is connected to two other network members, and these two members are also connected to each other, then alter A's clustering coefficient would be 1. Conversely, if none of the connections are mutually linked, the clustering coefficient score would be 0. The advantage of using the clustering coefficient is that it accounts for the local embeddedness of each alter and considers the potential clustering within the network. We focused on the clustering coefficient rather than alternative measures (e.g., local density) because it specifically captures the closure of triadic relationships, which is central to our theoretical conceptualization of embeddedness. While related measures of local cohesion may capture overlapping structural properties, they are not strictly equivalent, and the clustering coefficient provides a more direct operationalization of the mechanism of interest. In the models, we refer to that variable as *alter’s embeddedness*.

To address H2, which states that the behavior of ego is related to the composition within the cluster to which an alter belongs, we analyze the social domains of the individuals connected to the alter. Specifically, for each neighbor in the alter's network, we identify the social domain associated with that connection and calculate the diversity of these domains. For instance, if an alter is connected to four individuals, and all are categorized as “friends,” the social environment would be highly homogeneous. Conversely, if the connections are evenly distributed across different categories, the homogeneity would approach zero. To quantify this diversity, we calculate the heterogeneity index using the Index of Qualitative Variation (IQV) [38,73,74] at the node level. For H3, we will test the interaction of both the aforementioned constructs. That is, we will consider including an interaction term in the statistical model.

We include several control variables. First, we account for the dyad’s gender composition, as behavior differs depending on whether the speaking partner is of the same gender [75]. Women, in particular, may adapt more to their interlocutor [8] and produce fewer words in mixed-gender dyads, leading to increased word production by male counterparts [75]. A value of 1 indicates a same-gender dyad, while a value of 0 represents a mixed-gender dyad. Age is another factor, as linguistic variation is sensitive to life stage, particularly in adolescence and early adulthood [76]. We include a binary variable indicating whether ego and alter are in the same age group (1 = yes, 0 = no). We also include the social domain of the alter, whether it is a family member, a friend, or other, because friends and family are among the core social domains of influence in the identification process [50].

Additionally, we consider other relational dimensions, as convergence may occur through the frequency of interaction or the duration of the relationship. The more people interact and know each other, the more likely it is that they share similar practices [9,14,25]. Therefore, we include a variable that accounts for the number of years that ego and alter have known each other and one variable that accounts for the frequency of encounters, given by the question: ‘How often are you in contact with [alter’s name], whether face-to-face, by phone, internet, or another communication device? (5 = every day; 4 = almost every day; 3 = several times a week; 2 = several times per month; 1 = several times per year; 0 = less than several times per year).

### Analytical strategy

Our dataset includes repeated observations from the same respondents, which violates the assumption of independent observations. To account for this within-subject correlation, we employ statistical models designed to handle nested data structures and repeated measures. Specifically, we use Generalized Estimating Equations (GEE) [77], a population-averaged approach that estimates the average effect of predictors across the entire sample rather than focusing on individual-level (or subject-specific) variation, as is the case with multilevel models. GEE is particularly appropriate for our research aim, as we seek to understand overall behavioral patterns at the ego level rather than model variation in specific ego–alter dyads. In other words, GEE allows us to test whether a one-unit change in a predictor is associated with a change in the average outcome across the population, rather than assuming the same effect for each specific individual.

Given the guidelines of Arend and Schäfer (2019) [78], our sample size of both units of analysis (N = 21 n = 71) should provide sufficient statistical power to detect level-1 direct effects, considering the intraclass correlation (0.31) and the effect size of our different models (M1 conditional R^2^ = 0.38; M2 conditional R^2^= 0.39; M3 conditional R^2^ = 0.40). However, these conditional R^2^ values remain below the threshold typically required to reliably detect direct level-2 effects. For this reason, we do not interpret the coefficients of level-2 predictors but still include them as control variables in our models.

## Results

### Descriptive results: intensifier distribution

**Fig 1:**
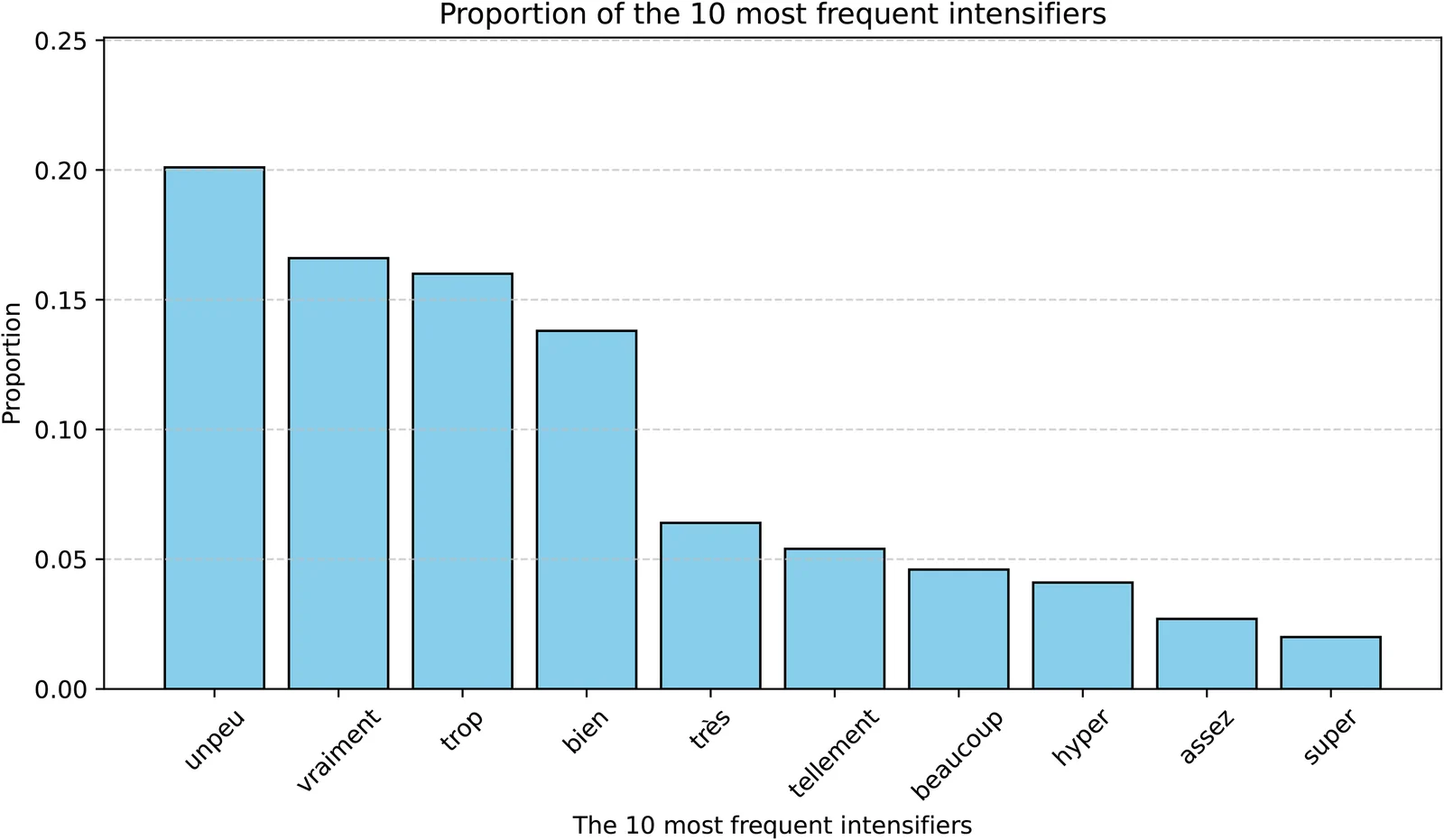
Distribution of the ten most frequent intensifiers (N = 2’055).

Fig 1 shows the distribution of the ten most frequent intensifiers in our corpus. In total, 2,055 intensifiers were retrieved. *Un peu* (‘a bit’) is the most frequently used intensifier by the speakers, accounting for 20%, followed by *vraiment* (‘really’) at 16.6%, and *trop* (‘so’) at 16%. The high frequency of *un peu* may be unexpected in the context of English intensifier studies, where *really*, *very*, and *so* are the most frequent even when downtoners (which decrease the degree of the following adjective) are included [60]. The reason for the dominance of *un peu* is that it often functions not strictly as a downtoner, but as a ‘mitigating’ device that carries pragmatic functions such as weakening the speaker’s commitment to the claim or indicating that the claim is the speaker’s subjective opinion. These are not unusual pragmatic functions for expressions that also carry independent semantic meaning; for example, *je pense que* ‘I think that’ fulfills similar pragmatic functions [79]. In the present data, these pragmatic functions of *un peu* can be seen most clearly in cases such as (1):

(1) je crois qu'elle a un peu rejeté ça **un peu** massivement

‘I think that she kind of rejected that **a bit** massively.’

In (1), the speaker is framing the claim as an opinion, as first indicated by *je crois* ‘I believe/think’. The second instance of *un peu* modifies *massivement* ‘massively’. In context, this instance of *un peu* cannot mean ‘a bit’ in the sense of ‘not very massively’ but rather signals mitigation of the claim, or acknowledgement of the subjectivity of the claim.

While some instances of *un peu* can be clearly understood as pragmatic markers, as in (1), the speaker’s intention in the majority of cases cannot be easily determined. For that reason, we include all instances and (as we show later) demonstrate that the results are robust to the exclusion of *un peu*.

The overall frequencies of intensifiers in Fig 1 also differ from those in Vargas Alvares (2024), an analysis of French intensifiers in 21st-century Quebecois films [63]. In the Quebecois data, *vraiment* is less frequent overall than *bien* and *très*, but among speakers between the ages of 5 and 17, *vraiment* is more frequent than *bien* and *très*. In this respect, the present data from Swiss French are consistent with the apparent intensifier change occurring in Quebecois French.

**Fig 2:**
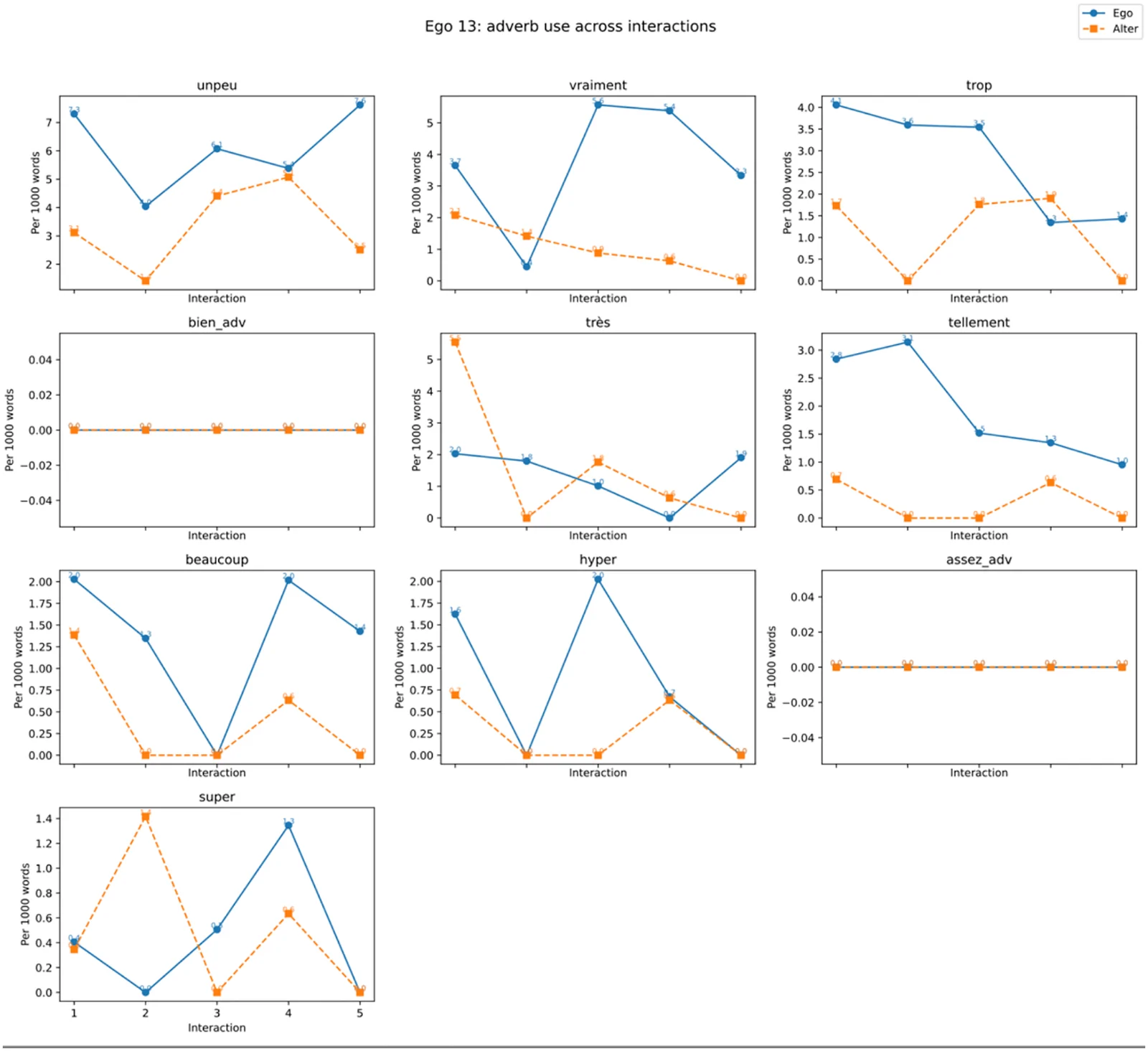
Example of the Top 10 intensifiers distribution for Ego 13 per interactions. **Recorded.** Note: On the x-axis, each value represents an interaction with a different alter.

Fig 2 illustrates how the behavior of one speaker may vary from one interaction to another, showing the frequencies of the ten most frequent intensifiers for both Ego13 (the focal speaker) and the alter in each interaction. Ego13 varies the frequency of intensifier use depending on both the specific intensifier and the interaction. We can also observe instances of convergence behavior, while in other interactions, the ego and alter differ in their intensifier usage patterns. For example, consider the intensifier *super*: the orange dot labeled “1” represents the frequency of *super* used by Ego13 in interaction with Alter1, while the blue dot represents the frequency with which Alter1 used *super* in the same interaction. We can see that Ego13 varies the occurrences of *super* across different interactions. Another example is *bien*, which Ego13 never used across all discussions; interestingly, none of the alters used this intensifier either. This provides further evidence that social mechanisms may underlie these behavioral patterns. The full distributions for each ego are available in the supplementary material.

We also examined (Table 1) whether intensifier distribution patterns vary under conditions of gender homophily (ego and alter being of the same gender) and age homophily (ego and alter belonging to the same age group). This analysis allows us to explore whether certain intensifiers are more likely to occur in specific types of interactions, and to assess potential relational asymmetries or power dynamics. In essence, we aim to determine whether the use of certain intensifiers is context-dependent, that is, influenced by situational or demographic factors, rather than reflecting genuine convergence behavior. If this were the case, the likelihood of observing a given intensifier would be constrained by contextual factors rather than by accommodation processes. Consequently, this could affect the Manhattan distance construct, as individual differences in intensifier usage might arise simply because certain intensifiers are unlikely to appear in particular conversational contexts by definition. Our results show no significant variation for either homophily measure. Moreover, the two tables display nearly identical distributions for age and gender homophily, suggesting that relational asymmetry is not driving the observed behavior. It is also worth noting that all ego–alter relationships analyzed represent close and significant ties, so we do not expect any formal speech or hierarchical differences between the egos and their alters. All conversations were inherently informal in nature.

**Table 1:** Intensifier distribution per homophily status.

	Un peu	Vraiment	Trop	Bien	Très	Tellement	Beaucoup	Hyper	Assez	Super
**Age Homophily**										
Yes	0.08	0.06	0.06	0.05	0.03	0.02	0.02	0.01	0.01	0.01
No	0.06	0.06	0.07	0.06	0.02	0.02	0.02	0.01	0.01	0.01
**Gender homophily**										
Yes	0.06	0.06	0.05	0.06	0.03	0.02	0.02	0.01	0.01	0.01
No	0.07	0.06	0.07	0.05	0.02	0.02	0.02	0.01	0.01	0.01

Note: Mean frequency per 1000 words.

Table 2 displays the distribution of our variables of interest. The dependent variable, convergence, has a mean of 4.21 with a standard deviation of 0.83, indicating moderate levels of convergence across participants. At the dyadic level, alter’s embeddedness has a mean of 0.62 (SD = 0.36), indicating moderate integration of alters within the network, while alter’s heterogeneity (mean = 0.43, SD = 0.45) shows considerable variation. Regarding dyadic characteristics, 54.93% of dyads share the same age, and 57.75% share the same gender. Relationships have varying durations (mean = 15.73, SD = 11.46) and frequencies of interaction (mean = 3.44, SD = 1.24). Social domain composition is mostly family (39.44%) and friends (39.44%), with a smaller portion of other relationships (21.13%). At the network level, density has a mean of 0.39 (SD = 0.20).

**Table 2:** Measurement distribution.

	Mean (Standard deviation)	Proportion
**Dependent variable**		
Convergence	4.21 (0.83)	
**Dyadic level (n = 71)**		
Alter’s embeddedness	0.62 (0.36)	
Alter’s heterogeneity	0.43 (0.45)	
Same age		54.93%
Same gender		57.75%
Duration of the relation	15.73 (11.46)	
Frequency of interactions	3.44 (1.24)	
Social domain Family Friends Other		39.44% 39.44% 21.13%
**Ego level (N = 21)**		
Density	0.39 (0.20)	
Gender (Ref. Female)		80.95%

Let us note that the Manhattan distance measure is strongly related to the choice of intensifiers on which our analyses are based. Therefore, the Manhattan score might be inconsistent if we modify the set of intensifiers, for instance, by adding or removing some of them. We have attempted to base our analyses on the most exhaustive list of intensifiers available to our knowledge. It is plausible that some intensifiers were not included, given their large variety. Moreover, we made a strong methodological claim by including *un peu*, a downtoner, in the analyses given that it carries multiple pragmatic functions. To ensure robustness, we conducted several additional checks to verify that our results do not depend on specific methodological choices. Specifically, we ran analyses excluding intensifiers with zero frequency across all egos and alters. We also computed the Manhattan distance using only the ten most frequent intensifiers. Lastly, we computed the Manhattan distance excluding *un peu*. As shown in Table 3, the correlation between the measures is high, indicating that the Manhattan distance remains stable regardless of the set of intensifiers included. Furthermore, for both robustness analyses, the regression results remain consistent in terms of the direction, magnitude, and significance of the effects.

**Table 3:** Pearson correlations for the different operationalizations of the Manhattan distance index.

Types of Manhattan distance measure	II. Without zero frequency	III. Top 10	IV. Without *un peu*
I. Original	0.99	0.97	0.96
II. Without zero frequency		0.97	0.96
III. Top 10			0.90

Note: I) The original Manhattan distance used in the analyses, (II) the Manhattan distance excluding intensifiers with zero frequency, (III) the Manhattan distance calculated with the ten most frequent intensifiers, (IV) the Manhattan distance without *un peu*.

### Regression results

Table 4 displays the pairwise correlation between the numeric variables. Table 5 indicates how an alter's network characteristics—specifically, alter heterogeneity, alter embeddedness, and their interaction—relate to linguistic convergence, the dependent variable in the regressions. Alter heterogeneity captures the extent to which an alter’s contacts come from varied social domains. In contrast, alter embeddedness reflects the degree to which an alter is situated in a structurally dense cluster, meaning that the alter’s contacts are closely interlinked.

In Model 1, the coefficient for alter embeddedness is positive and significant (0.58, p < 0.05). These results indicate that when an alter is embedded within a dense structural cluster, where their contacts are closely interconnected, linguistic similarity reaches a higher level. Model 2 includes alter's heterogeneity as the sole network predictor. The coefficient is in the expected direction but not statistically significant (−0.19, n.s.). Model 3 includes both predictors simultaneously. Overall, although there are minor changes in the effect size, the direction and significance of both variables remain consistent. These first sets of results therefore support H1 and lead us to reject H2.

**Table 4:** Pairwise Pearson correlations.

	1. Similarity	2.	3.	4.	5.	6.
2. Alter embeddedness	0.09					
3. Alter heterogeneity	0.12	−0.19				
4. Same age	0.01	0.09	0.17			
5. Same gender	−0.04	0.23*	0.02	−0.09		
6. Duration of the relation	0.05	0.18	−0.27*	−0.57***	0.22	
7. Frequency of interactions	0.11	0.03	0.05	0.05	−0.07	0.06

***p < 0.001**p < 0.01*p < 0.05

**Table 5:** GEE Models results for the association between the dyadic level variables and convergence (n = 71).

Variables	Model 1	Model 2	Model 3	Model 4
Intercept	4.07 (0.43)***	3.85 (0.57)***	3.86 (0.61)***	4.12 (0.44)***
*Alter’s relational characteristics*				
Alter embeddedness	0.58 (0.24)*		0.64 (0.23)**	0.65 (0.21)**
Heterogeneity of alter’s neighborhood		−0.19 (0.26)	−0.27 (0.24)	−0.24 (0.72)
Embeddedness x Alter Heterogeneity				−0.05 (1.1)
*Alter’s sociodemographic characteristics*				
Social domain (ref. Others) Family Friends	1.56 (0.73)* 0.48 (0.32)	1.30 (0.80) 0.58 (0.34)+	1.52 (0.76)* 0.47 (0.28)+	1.52 (0.87)* 0.47 (0.27)+
*Alter-ego relationship*				
Same age	0.20 (0.22)	0.23 (0.16)	0.21 (0.21)	0.21 (0.23)
Same gender	−0.55 (0.28)*	−0.47 (0.27)+	−0.60 (0.28)*	−0.60 (0.30)*
Duration of the relation	−0.04 (0.03)	−0.02 (0.03)	−0.04 (0.03)	−0.04 (0.03)
Frequency of interaction	0.10 (0.09)	0.11 (0.09)	0.08 (0.09)	0.09 (0.09)
*Ego characteristics*				
Gender (ref. Women) Men	−0.36 (0.39)	−0.34 (0.41)	−0.44 (0.40)	−0.44 (0.45)
Density of support	−1.22 (0.50)*	−0.78 (0.46)+	−1.12 (0.49)*	−1.11 (0.65)+

Note: Standard errors in parentheses; ***p ≤ 0.001 **p ≤ 0.01 *p ≤ 0.05 + p ≤ 0.1

In Model 4, both alter’s neighborhood heterogeneity and alter embeddedness are included alongside their interaction. In these interaction models, the main effect of alter embeddedness remains positive and becomes even slightly more pronounced, with a coefficient of 0.65 (p < 0.01). In this model, the main effect of alter embeddedness reflects its association with convergence when heterogeneity is equal to zero, that is, in fully homogeneous networks. Under these conditions, higher density is associated with increased convergence.

Conversely, the main effect of alter heterogeneity is absent in this model, with a coefficient of –0.24 (n.s.), suggesting that heterogeneity in a structurally sparse cluster does not significantly drive similarity.

The interaction term between alter embeddedness and alter heterogeneity is negative (–0.05), though not statistically significant. Although the interaction between density and heterogeneity does not reach conventional levels of statistical significance, the pattern of results remains consistent with our theoretical expectations. In particular, the positive main effect of cluster density indicates that alters embedded in denser local structures are associated with greater linguistic convergence when heterogeneity is minimal (i.e., in more homogeneous subnetworks). While we do not find strong statistical evidence that this effect varies significantly across levels of heterogeneity, the direction of the coefficients suggests that convergence is more likely under conditions combining structural embeddedness and compositional homogeneity. Taken together, these findings align with the theoretical expectations underlying H3. However, the statistical evidence does not allow us to firmly validate the hypothesis. This suggests that heterogeneity may play a more modest moderating role than anticipated, or that its influence may be better captured with larger samples or more fine-grained measures.

As for the other predictors, we observe that respondents seem to behave more similarly with family members (1.52, p < 0.05). To a lesser extent, people seem to use more similar language with friends than with people originating from other social domains (like colleagues, for instance). We observe a consistent effect of gender homophily across models (−0.60, p < 0.05), suggesting that when two interactants share the same gender, they are less likely to speak alike. The frequency and duration of the relation do not show any significant association, which goes against the basic intuition of the social influence process. However, as argued in the theoretical section, the mere frequency of interaction may lack the capacity to influence one’s behavior. Instead, what is exchanged in interaction seems to matter more. In our case, the fact that dense relations of support are related to similarity (−1.22, p < 0.05) might point in this direction. This effect suggests that when an ego is integrated in a network in which most of the contacts support each other, there is a tendency to show lesser similarity on average across interactions. This shifts the focus from the dyadic process to the importance of considering the meso-context and how the social space is organized around, and potentially even without, ego.

### Robustness Check

We acknowledge that the results may depend on how the similarity measure is operationalized and on specific methodological choices. To address this, we conducted additional analyses using the Manhattan distance to control for the potential influence of adverbs with zero occurrences. We also tested alternative specifications by restricting the analysis to the ten most frequent adverbs and by excluding “un peu,” which may be questionable as an adverb per se. Across these specifications, the measures remain highly correlated, and the results are substantively consistent.

That said, the results may also be sensitive to the choice of similarity measure. We retained the Manhattan distance because it is commonly used in natural language processing [71] studies; however, other measures are also widely employed. In particular, Euclidean distance, the Cosine, and Jaccard index are established approaches for assessing (dis)similarity. To evaluate the robustness of our findings, we conducted additional analyses using these alternative measures.

Because the Euclidean distance captures dissimilarity, we expect the direction of the coefficients to be reversed relative to the reversed Manhattan distance measure we used. The results based on Euclidean distance are consistent with our main findings, although the magnitude of the coefficient is slightly smaller (alter embeddedness: −0.22, p < 0.05). In contrast, the Jaccard index and the Cosine yield somewhat different results, although these do not fundamentally impede the interpretation of our findings. Specifically, models indicate a significant and negative coefficient for the heterogeneity of the alter’s neighborhood (Jaccard: −0.15, p < 0.05; Cosine: −0.28, p < 0.05), suggesting that when an alter originates from a structurally sparse and compositionally heterogeneous cluster, the ego is less likely to converge. Together, these robustness checks are consistent with our findings based on the Manhattan measure, as they allow us to draw similar tendencies from different perspectives.

## Discussion

Our results seem to indicate that the interaction between both structure (embeddedness of the alter) and composition (homogeneity of the alter’s social relations) is related to linguistic similarity at both the network and the dyadic level. Together, this aligns with the concept that for individuals to be able to manage effective social behavior and navigate their social environment, clear mental representations are important [37]. Our findings, therefore, seem to point in the direction that such mental representations are enhanced by clear structural and compositional factors.

At the dyadic level, we also observe that when the alter with which the ego interacts is embedded in a homogeneous and dense structural cluster, the respondent tends to align linguistically. In other words, a tightly knit, cohesive network around the alter seems to promote greater similarity in speech patterns between the ego and the alters. This reinforces the idea that being embedded in a structurally homogeneous network zone enhances linguistic similarity.

However, is the opposite also true? Although the estimates for heterogeneity of the alter’s contacts are not statistically significant, the direction of the coefficient suggests that when an alter is connected to others from varied social domains (a heterogeneous network), there may be a slight negative association with linguistic similarity. This pattern tentatively suggests that an alter who engages with a broad, diverse set of contacts who are not themselves tightly connected might not facilitate the ego’s adaptation of language to the same extent. The absence of significance in our models – potentially also due to the small sample size – does not allow us to draw conclusions in that sense, but a replication of this study with more data could help us determine if this claim holds. Typically, our robustness checks using different similarity measures point in this direction, providing additional confidence that future replication could confirm this intuition.

Although we cannot fully evaluate the effect of density due to the small sample size and potential lack of statistical power, we note that the direction of the density effect might appear counterintuitive at first, as the literature suggests that dense connections provide an environment favorable to developing shared linguistic norms [10,69]. According to this, we would have expected egos to become more similar to their alters. Nevertheless, a potential and plausible assumption for the direction of the density effect could be found in the faceted identity theory [54]. Within this framework, in dense heterogeneous networks, where individuals interact across diverse social roles, there is a greater likelihood of encountering differing norms and expectations. This increases the risk of social costs, as behaviors or language appropriate in one context may be inappropriate in another. To mitigate such risks, individuals tend to adopt a neutral, role-independent language style that facilitates communication across group boundaries. Yet, this would require further exploration with a larger sample size, to test whether or not this assumption holds.

Another result that caught our attention is the fact that ego seems to align linguistically more with family members. This is somewhat contrary to previous literature, indicating that young adults tend to distance themselves from family as they seek autonomy and strengthen peer affiliations, leading to reduced perceived linguistic similarity with parents [8,15,51]. This transition is marked by a linguistic shift known as the “adolescent peak” [8,40]. The contrast observed in our results may be attributable to the family members under study. Indeed, the cited references primarily focus on the parent–child dyad and on the distancing of young adults from their parents. In our sample, and in the recorded discussions, most of the family members were brothers and sisters, who might also be caught in overlapping relations, share cultural references, and be seen as peers. Together, these could explain the unexpected effect of social domains. This might call to differentiate family members and, more broadly, to go beyond the nuclear view of family as the only family structure [29]. Unfortunately, in the present paper, the sample size does not allow us to distinguish family members and test the differentiated effects. Further investigation is needed to determine whether this assumption holds or whether our results may instead reflect other statistical artifacts.

Lastly, we observed that gender homophily seems to restrain linguistic similarity. This might be surprising since we would expect people of the same gender to use similar language to acknowledge their belonging to the same social category [80]. A plausible assumption derived from the literature is that women, in particular, tend to accommodate more to their interlocutors [8] and produce fewer words in mixed-gender dyads, which often results in increased word production by their male counterparts [75]. Yet, the sample is highly biased toward women, and the negative effect could therefore be a mere artifact of our gender composition, where women tend to adapt more in mixed dyads. Consequently, same-gender dyads show less similarity compared to mixed ones, although the effect might differ in a more balanced sample.

### Limitations

This study has several limitations. First, we rely on cross-sectional data that prevent us from inferring causal mechanisms. Moreover, linguistic accommodation is acknowledged to be time-dependent [3,81], a process we cannot capture. For instance, Felder (2020) identified five distinct forms of accommodation from a study of WhatsApp conversations: convergence, divergence, independent variation, maintenance, and parallelism. It would therefore be interesting to reproduce this study with longitudinal data to see how the convergence dynamic is driven by network mechanisms. We can also imagine that convergence shapes networks and resource distribution among them, suggesting a reversed or cyclical process: structural and compositional properties may as well produce convergence, as convergence may foster tie creation [25].

In a similar vein, we have operationalized the convergence process by measuring how similar two individuals are. This approach might be limiting, because convergent behavior, as argued, may occur over time and even within a single conversation, with interlocutors gradually converging as the discussion unfolds. Because the linguistic variables under consideration are lexical and therefore discrete, it is not possible to directly observe convergence as a continuous, moment-to-moment adjustment, as is commonly done with phonetic features. Instead, our measure captures similarity across interactions, which does not fully disentangle static resemblance from dynamic adaptation. However, the observed within-speaker variation across interactional contexts suggests that speakers adjust their lexical behavior rather than relying on stable repertoires. In this sense, the measure used here can be interpreted as capturing convergence in an indirect but meaningful way. While future research could benefit from more fine-grained temporal data to better isolate dynamic processes (e.g., through priming-based or sequential analyses), we remain confident that the present approach provides a valid and informative approximation of convergence in the context of lexical variation.

We also acknowledge the lack of some potential key factors related to linguistic accommodation. For instance, we did not control for education, ethnicity, or other demographic factors. This stems mainly from the sample size and the lack of statistical power to include an extended set of variables. By focusing on gender and age, we aimed to control for some of the most striking variables identified in the field. Nevertheless, reproducing this research with additional variables would shed deeper light on the relationship between network mechanisms and language attuning.

Lastly, we want to remind readers of the exploratory nature of this paper. Because the study design was time-consuming for both researchers and participants, and given other temporal data collection constraints, we could not aim for a larger representative sample but had to rely on a quite small purposive sample. Nevertheless, this research is the first to collect both personal network data, including alter–alter ties, and face-to-face discussion data, at this scale. This study is therefore the first that allows us to explore how accommodation could be related to personal networks and relational processes, which we believe makes this research valuable. That said, we acknowledge that extending this study with additional data and a larger sample would strengthen the robustness of our results and help better disentangle the assumptions arising from our findings.

### Further directions

Some negative and quite surprising associations may be due to the fact that other processes of convergence are at play. For instance, divergence may also help the interaction and show a positive attitude toward the interlocutor to acknowledge their personality and their differences [9]. Therefore, divergence by means of differentiating the language or maintaining it may be a positive process rather than an expression of social rejection. Social rejection could also be of particular interest. Indeed, instead of studying positive ties, one could consider how negative ties (such as conflict) are involved in the convergence and divergence process.

CAT is a broad and complex theory that cannot be addressed within a single paper, as argued by some of the initiators of the theory [21]. Instead, one should focus on specific parts of the theory and try to elucidate empirical gaps. Further investigation should consider testing the network mechanisms with other speech indices. Indeed, accommodation is not unidimensional, and not all linguistic variables behave similarly in terms of convergence [93]. Considering other populations could also allow for a broader generalization of these findings.

## Conclusions

By integrating different relational levels (dyadic, cluster, and personal network), we have explored how social relations may play a role in the development of convergent behavior. Overall, these findings are preliminary, emphasizing the nuanced interplay between network structure and compositional diversity. An alter’s strong embeddedness in a cohesive cluster may be associated with greater linguistic convergence, promoting the alignment of speech between the ego and alters.

Our results raise questions about the role of density, which is often identified as central in norm enforcement. Indeed, the sociolinguistic literature, as well as social network theory, leads to the expectation that a dense network would foster linguistic similarity through norm enforcement. However, the fact that we do not observe this here prompts us to reconsider the ability of structural properties alone to influence individuals. Rather, it seems that these properties must be considered at a more local scale and in conjunction with network composition.
