# Supplementary material for: Embedded dyads: How the personal network relates to linguistic accommodation
Source: PLoS One. 2026 Sep 21;21(9):e0357457. doi: 10.1371/journal.pone.0357457 (PMC13592575; doi:10.1371/journal.pone.0357457)
Supplement: Supplementary file 1 [file pone.0357457.s002.docx]

Object: Re-submission of the article	*PONE-D-26-03386-3*

Dear Dr. Srebrenka Letina,

Please find enclosed the re-submission of the manuscript *“Embedded dyads: how the personal network relates to linguistic accommodation”,* which we submit for your exclusive consideration for *PLOS ONE*.

We are very grateful to the editor and the two additional anonymous reviewers for their thoughtful comments. We have addressed all points individually, as detailed in the responses below. The new modifications have been highlighted in yellow in the revised document. These changes have significantly clarified the content of the research and strengthened the argument, which we believe has improved the overall quality of the paper.

**Editorial requirements**

*Thank you for stating the following in the Acknowledgments Section of your manuscript:*

*“This research received no specific grant from any funding agency, commercial or not-for-profit sectors.”*

*We note that you have provided funding information that is not currently declared in your Funding Statement. However, funding information should not appear in the Acknowledgments section or other areas of your manuscript. We will only publish funding information present in the Funding Statement section of the online submission form.*

*Please remove any funding-related text from the manuscript and let us know how you would like to update your Funding Statement. Currently, your Funding Statement reads as follows:*

*“The author(s) received no specific funding for this work.”*

*Please include your amended statements within your cover letter; we will change the online submission form on your behalf.*

Every funding informations have been removed form the manuscript. We want to keep the same declaration : “The author(s) received no specific funding for this work.”

*Thank you for uploading your study's underlying data set. Unfortunately, the repository you have noted in your Data Availability statement does not qualify as an acceptable data repository according to PLOS's standards.*

*At this time, please upload the minimal data set necessary to replicate your study's findings to a stable, public repository (such as figshare or Dryad) and provide us with the relevant URLs, DOIs, or accession numbers that may be used to access these data. For a list of recommended repositories and additional information on PLOS standards for data deposition, please see https://journals.plos.org/plosone/s/recommended-repositories..*

The data are under embargo for a period of two years following the completion of the research project from which this article is derived, extending until 2028. This embargo cannot be modified, as it was specified in the original data management plan, submitted to the ethical committee.

Regarding the data repository, SWISSUbase (https://www.swissubase.ch/fr/) is widely used for social science and related research domains in Switzerland and complies with the FAIR principles. This repository was selected to align with the ethical requirements of the University of Geneva and the data management plan submitted to the University’s ethics committee (CUREG). Therefore, the choice of data repository cannot be modified.

*Please include your full ethics statement in the ‘Methods’ section of your manuscript file. In your statement, please include the full name of the IRB or ethics committee who approved or waived your study, as well as whether or not you obtained informed written or verbal consent. If consent was waived for your study, please include this information in your statement as well.*

It has been added in the method section.

*Please include a separate caption for each figure in your manuscript.*

We have added the figure caption within the text and uploaded the figure separately in the correct format.

*Please include a new copy of Table 1 in your manuscript; the current table is difficult to read. Please follow the link for more information:* [*https://journals.plos.org/plosone/s/tables*](https://journals.plos.org/plosone/s/tables)

Table 1 has been modified accordingly.

**Reviewer 1**

*The biggest issue is the very small sample size. With only 21 participants and 71 interactions, the statistical analyses are underpowered. This makes it difficult to draw strong conclusions from the results. In particular, some of the claims feel too confident. I would then suggest a more cautious interpretation of the results to strengthen the credibility of the paper.*

Thank you for pointing out this limitation. We agree that the claim may appear overstated in some instances, given the nature of the data and the relatively small sample size. The introduction, discussion, and conclusion have been revised to adopt a more exploratory tone.

*The conceptual framework is interesting but diffuse and not easy to read, especially for those outside this research area. I would then suggest clarifying better which mechanism is central to each hypothesis to make the argument more coherent.*

We thank the reviewer for this suggestion. We have substantially revised the manuscript to improve the clarity of the argument and strengthen the connections between the theoretical framework and the hypotheses. In particular, we have streamlined the theoretical section by removing concepts and discussions that did not directly contribute to the core argument. For instance, we have removed the section on homophily, which we felt did not add substantively to the paper.

We have also simplified the presentation of the hypotheses, notably by removing the paragraph on exchange networks, which we believe previously introduced unnecessary complexity and blurred the main argument. In addition, we have integrated key elements of the hypotheses more directly into the theoretical framework in order to introduce the core ideas earlier in the manuscript and improve the overall flow and coherence of the paper.

*There is some ambiguity in how network concepts are used. Terms like embeddedness, clustering, and density appear throughout the paper, but their distinctions are not always clearly maintained. If I understood well, embeddedness is operationalized using the clustering coefficient. It is not completely clear to me if you have used the global clustering coefficient or the transitivity of the network. Please specify better. Moreover, have you tried to use different measures of clustering? Are the results changing?*

We agree that some of the terminology was overlapping and potentially misleading. We have therefore revised the manuscript to systematize the terminology used to refer to “embeddedness” and ensure greater conceptual clarity.

Regarding the clustering coefficient, we did not rely on a global network measure. Instead, we constructed an alter-centered subnetwork for each alter and computed a local clustering coefficient at the alter level. This measure captures the extent to which an alter is embedded in a cohesive local structure, operationalized as the proportion of closed triads among its immediate neighbors, and is thus closely aligned with a transitivity-based perspective on embeddedness.

We focused on the clustering coefficient rather than alternative measures (e.g., local density) because it specifically captures the closure of triadic relationships, which is central to our theoretical conceptualization of embeddedness. While related measures of local cohesion may capture overlapping structural properties, they are not strictly equivalent, and the clustering coefficient provides a more direct operationalization of the mechanism of interest.

We have made it clearer in the method section.

*You have used the Manhattan distance to evaluate linguistic similarity. To check the robustness of the results, I think it is very important to compare the results using different types of similarity measures.*

Thank you for raising this issue. We have run robustness check with different (dis)similarity measures — jaccard index, euclidean distance, cosine – and all yield similar results. We have added a robustness check section.

Finally, we agree that some degree of covariation between men and women may exist, particularly among married individuals. We would expect such dependence to be most relevant in dyadic designs, where observations are drawn from identified pairs (e.g., partners or former partners). In the present study, however, the sampling procedure is based on a probabilistic design, which ensures—within reasonable limits—that observations are independent. Nevertheless, we acknowledge that some residual covariation or selection processes may still be present, and we explicitly address these possibilities in the limitations section.

*Finally, the paper would benefit from some improvements in writing and presentation. There are a number of small language issues and typos, and some sections of the theory feel repetitive. Tightening the prose and sharpening the link between hypotheses, measures, and results would make the argument easier to follow.*

Thank you for the suggestion. The manuscript has been thoroughly revised and proofread, and we have worked to streamline the argument and remove repetitive and unclear sections.

**Reviewer 2**

*First, the empirical basis of the study is quite limited. The analysis relies on a small sample, with a highly unbalanced gender distribution. While the authors acknowledge these constraints, the strength of the conclusions at times exceeds what such a dataset can robustly support. In particular, with only 21 clusters, the reliability of higher-level effects and interaction terms is necessarily limited. The paper should be more explicitly framed as exploratory and hypothesis-generating rather than confirmatory.*

Thank you for raising this concern. We agree that this is a key limitation of our paper. As mentioned in our response to a similar comment by Reviewer 1, we acknowledge that some of our claims may have appeared overstated, given the nature of the data and the relatively small sample size. We have therefore revised the introduction, discussion, and conclusion to adopt a more exploratory and cautious tone.

*Second, the interpretation of the interaction hypothesis (H3) is problematic. The interaction term between embeddedness and heterogeneity is not statistically significant, yet the manuscript concludes that H3 is supported. This is not methodologically justified. At best, the results are inconclusive with respect to the interaction effect, and the conclusions should be revised accordingly.*

We thank the reviewer for this important clarification. We agree that the lack of statistical significance of the interaction term does not allow us to claim full empirical support for H3. In response, we have revised the manuscript to soften our interpretation and avoid overstating the findings.

Specifically, we now frame the results as providing partial and suggestive support for the hypothesized relationship, rather than definitive evidence of an interaction effect. As clarified in the revised text, while the interaction between density and heterogeneity does not reach conventional levels of statistical significance, the direction of the coefficients remains consistent with our theoretical expectations. In particular, the positive main effect of density indicates that convergence is more likely in structurally dense and compositionally homogeneous contexts (i.e., when heterogeneity is minimal).

However, we now explicitly acknowledge that we do not find strong statistical evidence that this relationship varies significantly across levels of heterogeneity. Accordingly, we have revised both the results and discussion sections to present H3 as not formally supported, but directionally consistent with the theoretical argument, and we emphasize the need for further research to more conclusively assess the moderating role of heterogeneity.

*Third, the operationalization of accommodation raises some concerns. The dependent variable captures overall linguistic similarity across interactions rather than dynamic accommodation as it unfolds within conversation. As a result, it is difficult to distinguish convergence from simple similarity or shared topics. The authors acknowledge this but this deserves stronger emphasis, as it directly affects the interpretation of the findings*

We thank the reviewer for this important point, which highlights a key conceptual distinction between convergence and similarity. We agree that convergence is most straightforward to observe when linguistic variables are continuous—such as pitch, rhythm, or vowel formants—where gradual adjustments can be tracked directly over time. In contrast, lexical variables are inherently discrete, which makes the direct observation of convergence dynamics more challenging.

In the case of lexical choices, capturing fine-grained temporal convergence would require additional assumptions, for instance regarding priming effects based on recently used items. However, such dynamics are not directly observable in the present data. For this reason, our approach focuses on variation across interactions rather than within-interaction temporal sequences.

Although our analysis does not explicitly disentangle convergence in a strictly dynamic sense from similarity in a static sense, it nevertheless captures systematic within-speaker variation across interactional contexts. In other words, speakers do not exhibit fixed lexical profiles but adjust their usage depending on the interaction. We interpret these interaction-dependent shifts as indicative of convergence processes, even if they are not measured at a micro-temporal level.

From this perspective, relying solely on similarity would risk overlooking the adaptive nature of linguistic behavior by implicitly assuming stability across contexts. By contrast, our approach allows us to capture meaningful changes in speakers’ lexical patterns across interactions, which we argue provides indirect but substantively relevant evidence of convergence.

A paragraph highlighting this point has been added to the limitations section.

*Fourth, some interpretations appear post hoc or somewhat overstated, especially in relation to unexpected findings (e.g., density effects, family effects). While these interpretations are plausible, they should be presented more cautiously given the limited statistical power.*

We have softened our claim and reframed it as a theoretically grounded assumption rather than a definitive conclusion.

We would like to thank you in advance for considering this research for publication and look forward to hearing from you at your earliest convenience

Yours sincerely,

Guillaume P. Fernandez
