# Supplementary material for: Embedded dyads: How the personal network relates to linguistic accommodation
Source: PLoS One. 2026 Sep 21;21(9):e0357457. doi: 10.1371/journal.pone.0357457 (PMC13592575; doi:10.1371/journal.pone.0357457)
Supplement: Peer review history

# Peer review: Decision Letter 0

**DOI:** 10.1371/journal.pone.0357457.r001

**Authors:** Srebrenka Letina

30 Mar 2026

Dear Dr. Fernandez,

Thank you for submitting your manuscript to PLOS ONE. After careful consideration, we feel that it has merit but does not fully meet PLOS ONE’s publication criteria as it currently stands. Therefore, we invite you to submit a revised version of the manuscript that addresses the points raised during the review process.

Both reviewers find the topic interesting and the integration of social network structure with linguistic accommodation promising. However, they also raise several important concerns that need to be addressed before the manuscript can be considered for publication.

In particular, the reviewers highlight limitations related to the small sample size, the interpretation of the interaction hypothesis, the operationalization of accommodation, and the need for more cautious and exploratory framing of the findings. They also suggest clarifying network measures, strengthening robustness checks, and improving the clarity of the manuscript.

In light of these comments, I invite you to revise the manuscript and resubmit it for further consideration. Please provide a detailed response to the reviewers outlining how each point has been addressed.

Please submit your revised manuscript by May 14 2026 11:59PM. If you will need more time than this to complete your revisions, please reply to this message or contact the journal office at plosone@plos.org. When you're ready to submit your revision, log on to https://www.editorialmanager.com/pone/ and select the 'Submissions Needing Revision' folder to locate your manuscript file.

- A letter that responds to each point raised by the academic editor and reviewer(s). You should upload this letter as a separate file labeled 'Response to Reviewers'.
- A marked-up copy of your manuscript that highlights changes made to the original version. You should upload this as a separate file labeled 'Revised Manuscript with Track Changes'.
- An unmarked version of your revised paper without tracked changes. You should upload this as a separate file labeled 'Manuscript'.

If you would like to make changes to your financial disclosure, please include your updated statement in your cover letter. Guidelines for resubmitting your figure files are available below the reviewer comments at the end of this letter.

If applicable, we recommend that you deposit your laboratory protocols in protocols.io to enhance the reproducibility of your results. Protocols.io assigns your protocol its own identifier (DOI) so that it can be cited independently in the future. For instructions see: https://journals.plos.org/plosone/s/submission-guidelines#loc-laboratory-protocols. Additionally, PLOS ONE offers an option for publishing peer-reviewed Lab Protocol articles, which describe protocols hosted on protocols.io. Read more information on sharing protocols at https://plos.org/protocols?utm_medium=editorial-email&utm_source=authorletters&utm_campaign=protocols.

As the corresponding author, your ORCID iD is verified in the submission system and will appear in the published article. PLOS supports the use of ORCID, and we encourage all coauthors to register for an ORCID iD and use it as well. Please encourage your coauthors to verify their ORCID iD within the submission system before final acceptance, as unverified ORCID iDs will not appear in the published article. *Only*  the individual author can complete the verification step; PLOS staff *cannot*  verify ORCID iDs on behalf of authors.

We look forward to receiving your revised manuscript.

Kind regards,

Srebrenka Letina, Ph.D.

Academic Editor

PLOS One

Journal Requirements:

When submitting your revision, we need you to address these additional requirements.

1. Please ensure that your manuscript meets PLOS ONE's style requirements, including those for file naming. The PLOS ONE style templates can be found at

https://journals.plos.org/plosone/s/file?id=wjVg/PLOSOne_formatting_sample_main_body.pdf and

https://journals.plos.org/plosone/s/file?id=ba62/PLOSOne_formatting_sample_title_authors_affiliations.pdf

2. Thank you for stating the following in the Acknowledgments Section of your manuscript:

“This research received no specific grant from any funding agency, commercial or not-for-profit sectors.”

We note that you have provided funding information that is not currently declared in your Funding Statement. However, funding information should not appear in the Acknowledgments section or other areas of your manuscript. We will only publish funding information present in the Funding Statement section of the online submission form.

Please remove any funding-related text from the manuscript and let us know how you would like to update your Funding Statement. Currently, your Funding Statement reads as follows:

“The author(s) received no specific funding for this work.”

Please include your amended statements within your cover letter; we will change the online submission form on your behalf.

3. Thank you for uploading your study's underlying data set. Unfortunately, the repository you have noted in your Data Availability statement does not qualify as an acceptable data repository according to PLOS's standards.

At this time, please upload the minimal data set necessary to replicate your study's findings to a stable, public repository (such as figshare or Dryad) and provide us with the relevant URLs, DOIs, or accession numbers that may be used to access these data. For a list of recommended repositories and additional information on PLOS standards for data deposition, please see https://journals.plos.org/plosone/s/recommended-repositories.

4. Please include your full ethics statement in the ‘Methods’ section of your manuscript file. In your statement, please include the full name of the IRB or ethics committee who approved or waived your study, as well as whether or not you obtained informed written or verbal consent. If consent was waived for your study, please include this information in your statement as well.

5. Please include a separate caption for each figure in your manuscript.

6. Please include a new copy of Table 1 in your manuscript; the current table is difficult to read. Please follow the link for more information: https://journals.plos.org/plosone/s/tables

7. If the reviewer comments include a recommendation to cite specific previously published works, please review and evaluate these publications to determine whether they are relevant and should be cited. There is no requirement to cite these works unless the editor has indicated otherwise.

[Note: HTML markup is below. Please do not edit.]

Reviewers' comments:

Reviewer's Responses to Questions

**Comments to the Author**

1. Is the manuscript technically sound, and do the data support the conclusions?

Reviewer #1: Partly

Reviewer #2: Partly

**********

2. Has the statistical analysis been performed appropriately and rigorously?

Reviewer #1: Yes

Reviewer #2: Yes

**********

3. Have the authors made all data underlying the findings in their manuscript fully available?

The PLOS Data policy

Reviewer #1: No

Reviewer #2: No

**********

4. Is the manuscript presented in an intelligible fashion and written in standard English?

Reviewer #1: Yes

Reviewer #2: Yes

**********

Reviewer #1: I found this paper interesting since it tries to include social network structure in computational linguistics studies.

These are my suggestions to improve the paper:

- The biggest issue is the very small sample size. With only 21 participants and 71 interactions, the statistical analyses are underpowered. This makes it difficult to draw strong conclusions from the results. In particular, some of the claims feel too confident. I would then suggest a more cautious interpretation of the results to strengthen the credibility of the paper.

- The conceptual framework is interesting but diffuse and not easy to read, especially for those outside this research area. I would then suggest clarifying better which mechanism is central to each hypothesis to make the argument more coherent.

- There is some ambiguity in how network concepts are used. Terms like embeddedness, clustering, and density appear throughout the paper, but their distinctions are not always clearly maintained. If I understood well, embeddedness is operationalized using the clustering coefficient. It is not completely clear to me if you have used the global clustering coefficient or the transitivity of the network. Please specify better. Moreover, have you tried to use different measures of clustering? Are the results changing?

- You have used the Manhattan distance to evaluate linguistic similarity. To check the robustness of the results, I think it is very important to compare the results using different types of similarity measures.

- Finally, the paper would benefit from some improvements in writing and presentation. There are a number of small language issues and typos, and some sections of the theory feel repetitive. Tightening the prose and sharpening the link between hypotheses, measures, and results would make the argument easier to follow.

Reviewer #2: The manuscript proposes an interesting and theoretically coherent extension of Communication Accommodation Theory by incorporating personal network structure into the analysis of linguistic convergence. The integration of sociolinguistic theory with social network analysis is well motivated, and the effort to combine personal network data with recorded interactions is methodologically innovative. The paper is clearly written, logically structured, and overall easy to follow.

However, there are important limitations that need to be addressed before the manuscript can be considered for publication.

First, the empirical basis of the study is quite limited. The analysis relies on a small sample, with a highly unbalanced gender distribution. While the authors acknowledge these constraints, the strength of the conclusions at times exceeds what such a dataset can robustly support. In particular, with only 21 clusters, the reliability of higher-level effects and interaction terms is necessarily limited. The paper should be more explicitly framed as exploratory and hypothesis-generating rather than confirmatory.

Second, the interpretation of the interaction hypothesis (H3) is problematic. The interaction term between embeddedness and heterogeneity is not statistically significant, yet the manuscript concludes that H3 is supported. This is not methodologically justified. At best, the results are inconclusive with respect to the interaction effect, and the conclusions should be revised accordingly.

Third, the operationalization of accommodation raises some concerns. The dependent variable captures overall linguistic similarity across interactions rather than dynamic accommodation as it unfolds within conversation. As a result, it is difficult to distinguish convergence from simple similarity or shared topics. The authors acknowledge this but this deserves stronger emphasis, as it directly affects the interpretation of the findings.

Fourth, some interpretations appear post hoc or somewhat overstated, especially in relation to unexpected findings (e.g., density effects, family effects). While these interpretations are plausible, they should be presented more cautiously given the limited statistical power.

I would recommend major revision, with particular attention to (i) reframing the study as exploratory, (ii) revising the interpretation of H3, (iii) clarifying the limits of the similarity measure, and (iv) strengthening the discussion of methodological constraints.

**********

what does this mean?). If published, this will include your full peer review and any attached files.

If you choose “no”, your identity will remain anonymous but your review may still be made public.

**Do you want your identity to be public for this peer review?**  For information about this choice, including consent withdrawal, please see our Privacy Policy

Reviewer #1: No

Reviewer #2: No

**********

[NOTE: If reviewer comments were submitted as an attachment file, they will be attached to this email and accessible via the submission site. Please log into your account, locate the manuscript record, and check for the action link "View Attachments". If this link does not appear, there are no attachment files.]

To ensure your figures meet our technical requirements, please review our figure guidelines: https://journals.plos.org/plosone/s/figures

You may also use PLOS’s free figure tool, NAAS, to help you prepare publication quality figures: https://journals.plos.org/plosone/s/figures#loc-tools-for-figure-preparation.

NAAS will assess whether your figures meet our technical requirements by comparing each figure against our figure specifications.

# Author comment: Author response to Decision Letter 1

**DOI:** 10.1371/journal.pone.0357457.r002

18 Jun 2026

All responses to reviewers have been included in the document called Revision cover letter PlosOne.docx

Attachment Submitted filename: Response to reviewers PONE-D-26-03386.docx

# Peer review: Decision Letter 1

**DOI:** 10.1371/journal.pone.0357457.r003

**Authors:** Claudia Noemi González Brambila

19 Aug 2026

Embedded dyads: how the personal network relates to linguistic accommodation

PONE-D-26-03386R1

Dear Dr. Fernandez,

We’re pleased to inform you that your manuscript has been judged scientifically suitable for publication and will be formally accepted for publication once it meets all outstanding technical requirements. Please, look at the suggestion reviewer 2 is made.

Within one week, you’ll receive an e-mail detailing the required amendments. When these have been addressed, you’ll receive a formal acceptance letter and your manuscript will be scheduled for publication.

An invoice will be generated when your article is formally accepted. Please note, if your institution has a publishing partnership with PLOS and your article meets the relevant criteria, all or part of your publication costs will be covered. Please make sure your user information is up-to-date by logging into Editorial Manager at Editorial Manager® and clicking the ‘Update My Information' link at the top of the page. For questions related to billing, please contact billing support.

If your institution or institutions have a press office, please notify them about your upcoming paper to help maximize its impact. If they’ll be preparing press materials, please inform our press team as soon as possible -- no later than 48 hours after receiving the formal acceptance. Your manuscript will remain under strict press embargo until 2 pm Eastern Time on the date of publication. For more information, please contact onepress@plos.org.

Kind regards,

Claudia Noemi González Brambila, Ph.D.

Academic Editor

PLOS One

Additional Editor Comments (optional):

Reviewers' comments:

Reviewer's Responses to Questions

**Comments to the Author**

Reviewer #1: All comments have been addressed

Reviewer #2: All comments have been addressed

**********

2. Is the manuscript technically sound, and do the data support the conclusions?

Reviewer #1: Yes

Reviewer #2: Yes

**********

3. Has the statistical analysis been performed appropriately and rigorously?

Reviewer #1: Yes

Reviewer #2: Yes

**********

4. Have the authors made all data underlying the findings in their manuscript fully available?

The PLOS Data policy

Reviewer #1: No

Reviewer #2: No

**********

5. Is the manuscript presented in an intelligible fashion and written in standard English?

Reviewer #1: Yes

Reviewer #2: Yes

**********

Reviewer #1: (No Response)

Reviewer #2: I have only one remaining suggestion. Although the discussion of H3 has been substantially improved, I would still encourage the authors to be particularly cautious when referring to "partial support" for this hypothesis. Since the interaction term is not statistically significant, it may be preferable to state that the observed pattern is consistent with the theoretical expectation, while emphasizing that direct statistical evidence for the hypothesized interaction remains inconclusive. This is, however, a minor point that does not affect my overall recommendation for publication.

**********

what does this mean?). If published, this will include your full peer review and any attached files.

If you choose “no”, your identity will remain anonymous but your review may still be made public.

**Do you want your identity to be public for this peer review?**  For information about this choice, including consent withdrawal, please see our Privacy Policy

Reviewer #1: No

Reviewer #2: No

**********

# Editor report: Acceptance letter

**DOI:** 10.1371/journal.pone.0357457.r004

**Authors:** Claudia Noemi González Brambila

PONE-D-26-03386R1

PLOS One

Dear Dr. Fernandez,

I'm pleased to inform you that your manuscript has been deemed suitable for publication in PLOS One. Congratulations! Your manuscript is now being handed over to our production team.

At this stage, our production department will prepare your paper for publication. This includes ensuring the following:

* All references, tables, and figures are properly cited

* All relevant supporting information is included in the manuscript submission,

* There are no issues that prevent the paper from being properly typeset

You will receive further instructions from the production team, including instructions on how to review your proof when it is ready. Please keep in mind that we are working through a large volume of accepted articles, so please give us a few days to review your paper and let you know the next and final steps.

Lastly, if your institution or institutions have a press office, please let them know about your upcoming paper now to help maximize its impact. If they'll be preparing press materials, please inform our press team within the next 48 hours. Your manuscript will remain under strict press embargo until 2 pm Eastern Time on the date of publication. For more information, please contact onepress@plos.org.

You will receive an invoice from PLOS for your publication fee after your manuscript has reached the completed accept phase. If you receive an email requesting payment before acceptance or for any other service, this may be a phishing scheme. Learn how to identify phishing emails and protect your accounts at https://explore.plos.org/phishing.

If we can help with anything else, please email us at customercare@plos.org.

Thank you for submitting your work to PLOS One and supporting open access.

Kind regards,

PLOS One Editorial Office Staff

on behalf of

Dr. Claudia Noemi González Brambila

Academic Editor

PLOS One
